# Correction: A comprehensive apoptotic assessment of niloticin in cervical cancer cells: a tirucallane-type triterpenoid from *Aphanamixis polystachya* (Wall.) Parker

**DOI:** 10.1039/d4md90049a

**Published:** 2024-12-02

**Authors:** Anuja Gracy Joseph, Mohanan Biji, Vishnu Priya Murali, Daisy R. Sherin, Alisha Valsan, Vimalkumar P. Sukumaran, Kokkuvayil Vasu Radhakrishnan, Kaustabh Kumar Maiti

**Affiliations:** a Chemical Sciences and Technology Division (CSTD), Organic Chemistry Section, CSIR-National Institute for Interdisciplinary Science and Technology (CSIR-NIIST) Industrial Estate Thiruvananthapuram 695019 India radhu2005@gmail.com kkmaiti@niist.res.in; b Academy of Scientific and Innovative Research (AcSIR) Ghaziabad 201002 India; c School of Digital Sciences, Kerala University of Digital Sciences, Innovation and Technology Thiruvananthapuram-695317 India

## Abstract

Correction for ‘A comprehensive apoptotic assessment of niloticin in cervical cancer cells: a tirucallane-type triterpenoid from *Aphanamixis polystachya* (Wall.) Parker’ by Anuja Gracy Joseph *et al.*, *RSC Med. Chem.*, 2024, **15**, 3444–3459, https://doi.org/10.1039/D4MD00318G.

The authors regret the omission of one of the affiliations for authors Kokkuvayil Vasu Radhakrishnan and Kaustabh Kumar Maiti from the original manuscript. The corrected list of authors and affiliations for this paper is shown above.

The Royal Society of Chemistry apologises for these errors and any consequent inconvenience to authors and readers.

